# Medicaid Enrollment After Hospital Presumptive Eligibility in the Emergency Department

**DOI:** 10.1001/jamahealthforum.2025.0768

**Published:** 2025-04-25

**Authors:** Simeng Wang, Katherine Arnow, Mason M. Sakamoto, Lisa Marie Knowlton

**Affiliations:** 1 Department of Surgery, Stanford University School of Medicine, Stanford, California; 2 Stanford University School of Medicine, Stanford, California; 3 Stanford-Surgery Policy Improvement Research and Education Center (S-SPIRE), Stanford, California

## Abstract

**Importance:**

Hospital presumptive eligibility (HPE) is an emergency Medicaid program enabling eligible uninsured patients to temporarily access Medicaid benefits during hospital encounters. It provides a pathway to long-term Medicaid coverage, but this feature may be underutilized among patients who received HPE in the emergency department (ED) and were discharged immediately thereafter.

**Objective:**

To characterize if 6-month Medicaid enrollment rates varied by patient demographics and ED encounter characteristics and identify factors that potentially impact enrollment.

**Design, Setting, and Participants:**

A retrospective cohort study analyzing data from HPE-participating EDs in California between January 1, 2016, and December 31, 2021, was carried out from January 2024 to November 2024. Unadjusted differences in 6-month Medicaid enrollment rates among patient groups were analyzed with χ^2^ tests. Multivariable logistic regression was used to evaluate the adjusted odds of enrollment by different demographic and encounter characteristics. Patients aged 19 to 64 years who were uninsured, were treated and released from ED, and received HPE during the encounter were included.

**Exposures:**

Receiving HPE during ED encounters.

**Main Outcomes:**

The primary outcome was Medicaid enrollment within 6 months after receiving HPE in ED.

**Results:**

Of the 585 693 patients who received HPE during ED treat-and-release encounters, 175 495 were of Hispanic ethnicity (30.0%), 73 518 were White (12.6%), 33 829 were Black (5.8%), 12 824 were Asian or Pacific Islanders (2.2%), 1685 were Alaskan Native or American Indian (0.003%), 27 610 were of other race and ethnicity (0.05%), and 260 732 did not report race and ethnicity (44.5%). A total of 217 430 (37.1%) of 585 693 patientsof the total study population who received HPE during ED treat-and-release encounters enrolled in Medicaid by 6 months. In the regression model, male (adjusted odds ratio [aOR] 0.74; 95% CI, 0.72-0.76; *P *< .001), Hispanic (vs White: aOR, 0.94; 95% CI, 0.90-0.98; *P* = .007), and Spanish speaking (vs English speaking: aOR, 0.72; 95% CI, 0.68-0.77; *P* < .001) patients were less likely to enroll in Medicaid coverage. Annual enrollment rates declined notably following the onset of the COVID-19 pandemic (40.6% to 29.8%). Weekend encounters were more likely to have lower enrollment (vs weekday: aOR, 0.95; 95% CI, 0.93-0.97; *P* < .001). EDs in public hospitals (vs nonprofit: aOR, 1.27; 95% CI, 1.04-1.55; *P* = .02) or large hospitals (vs small: aOR, 1.13; 95% CI, 1.00-1.27; *P* = .04) were more likely to have higher enrollment. There was variable enrollment across different regions of California (27.3%-47.2%).

**Conclusions and Relevance:**

In this cohort study, Medicaid enrollment rates after receiving HPE in ED varied across different patient, facility, and geographic characteristics, highlighting the need for additional resources to ensure Medicaid coverage among this high-risk uninsured population.

## Key Points

**Question:**

Do 6-month Medicaid enrollment rates after receiving hospital presumptive eligibility (HPE) in the emergency department (ED) vary by patient demographic and clinical characteristics?

**Findings:**

In this cohort study of 585 693 patients, 217 430 (37.1%) who received HPE during ED treat-and-release encounters in California from 2016 to 2021 enrolled in Medicaid within 6 months. Male sex, Hispanic ethnicity, Spanish-preferred language, and weekend encounters were among the factors associated with significantly lower odds of enrollment; in addition, decreased enrollment was observed following the onset of the COVID-19 pandemic and geographic variability in California.

**Meaning:**

The findings of this study underscore disparities in Medicaid enrollment after receiving HPE at the time of ED encounter, highlighting potential barriers that can be addressed to expand long-term Medicaid coverage among uninsured patients.

## Teaser

This cohort study characterizes the association of 6-month Medicaid enrollment rates with patient demographics and emergency department encounter characteristics and identifies factors that may impact enrollment.

## Introduction

For 27.1 million uninsured individuals in the US, the emergency department (ED) is the safety net for primary, specialty, and lifesaving care.^1^ ED costs can result in catastrophic health expenditures.^2,3^ It is critical to identify resources for uninsured patients that minimize financial burden and promote access.

Hospital presumptive eligibility (HPE) was implemented in 2014 as a part of the Affordable Care Act that aims at decreasing out-of-pocket costs and increasing appropriate health care utilization among uninsured patients. It allows qualified individuals to temporarily access Medicaid benefits for up to 60 days and offers a pathway to long-term Medicaid coverage. Hospital staff assist HPE recipients with full-scope Medicaid applications so they can enroll in Medicaid after HPE expires.^4,5^ Prior studies have demonstrated that 65% of inpatient recipients enroll in Medicaid by 6 months after HPE, suggesting its role as a bridge to long-term coverage.^6,7^ However, there is a paucity of literature on HPE patients discharged from the ED. In this study, we queried statewide claims and eligibility data to delineate the patterns of Medicaid enrollment among ED-discharged HPE patients, hypothesizing that enrollment rates are low overall due to variable demographic and utilization characteristics.

## Methods

We retrospectively analyzed Medicaid claims and enrollment data from the California Department of Health Care Services (DHCS) (January 1, 2016-December 31, 2021). Patients aged 19 to 64 years who received HPE were included and sorted into the ED cohort or inpatient cohort based on the claim type. We excluded patients who died, had HPE coverage for more than 6 months, received more than 1 HPE annually before 2020, or more than 2 annually from 2020 to 2021 (DHCS expanded HPE to 2 eligible periods per year in 2020).^8^ For each cohort, the primary outcome was Medicaid enrollment by 6 months. The between-cohort differences were compared with χ^2^ tests. For the ED cohort, unadjusted differences in enrollment were analyzed with χ^2^ tests. Multivariable logistic regression evaluated adjusted odds of enrollment (variables are listed in eTable 4 in Supplement 1). A 2-tailed *P* < .05 was the threshold for statistical significance. Statistical analyses were performed using Stata MP statistical software (version 16, StataCorp). The study was reported in concordance with the Strengthening the Reporting of Observational Studies in Epidemiology (STROBE) reporting guidelines for cohort studies. This study was approved by the DHCS and the institutional review board of Stanford University School of Medicine.

## Results

**Figure 1.**
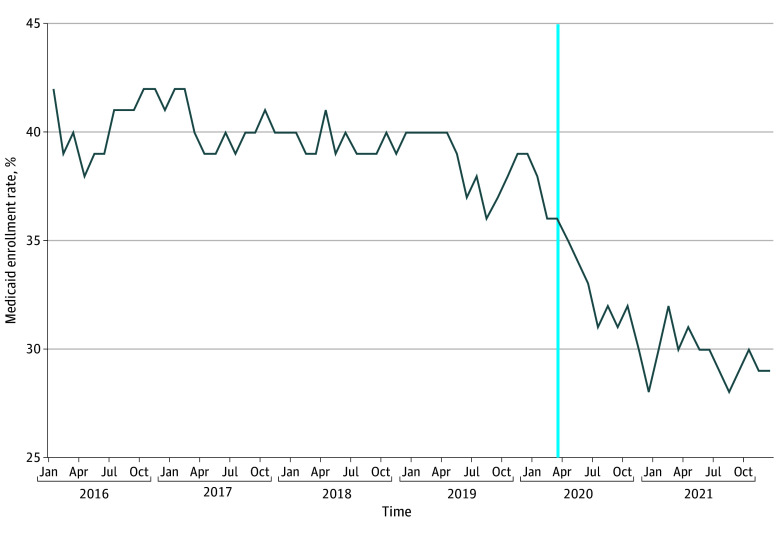
Medicaid Enrollment Rate by 6 Months After Receiving Hospital Presumptive Eligibility (HPE) in the Emergency Department (ED) (January 2016-December 2021) The blue line marks the beginning of the COVID-19 pandemic and specifically when shutdowns started to be implemented. ED indicates emergency department; HPE, hospital presumptive eligibility.

**Figure 2.**
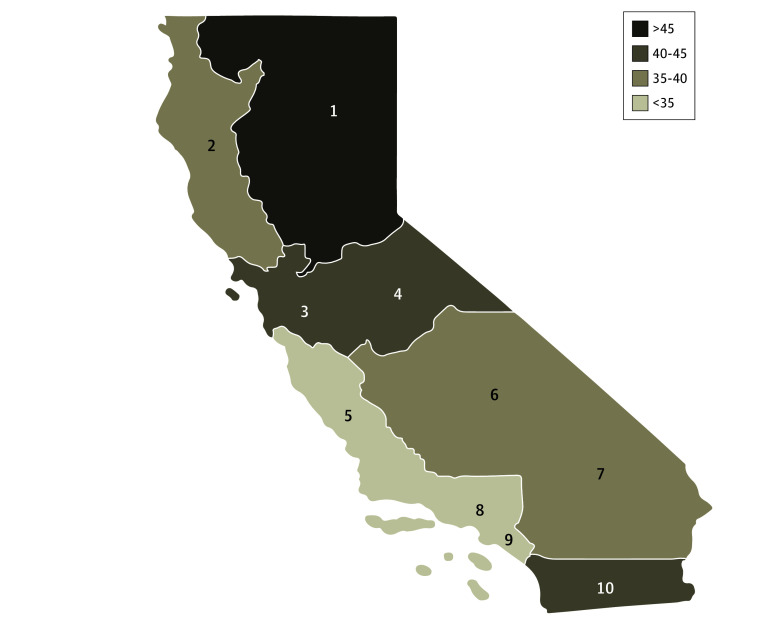
Medicaid Enrollment Rate by 6 Months After Receiving Hospital Presumptive Eligibility (HPE) in the Emergency Department (ED) by Regions of California Regional numbers: (1) Superior California, (2) North Coast, (3) San Francisco Bay Area, (4) Northern San Joaquin Valley, (5) Central Coast, (6) Southern San Joaquin Valley, (7) Inland Empire, (8) Los Angeles County, (9) Orange County, and (10) San Diego–Imperial.

Claims from 585 693 ED encounters were analyzed after inclusion and exclusion criteria were applied (eFigure in Supplement 1). 175 495 were of Hispanic ethnicity (30.0%), 73 518 were White (12.6%), 33 829 were Black (5.8%), 12 824 were Asian or Pacific Islanders (2.2%), 1685 were Alaskan Native or American Indian (0.003%), 27 610 were of other race and ethnicity (0.05%), and 260 732 did not report race and ethnicity (44.5%). A total of 217 430 ED HPE recipients (37.1%) enrolled in Medicaid by 6 months. The unadjusted differences in enrollment among different patient and encounter subgroups are reported in eTable 1 in Supplement 1. Male patients had lower enrollment rates than female patients (32.8% vs 43%), as did Spanish speakers (27.6%) compared with patients who speak other languages. Patients who did not disclose race and ethnicity or their preferred language had the lowest enrollment rates (5.2% and 3.9%, respectively). Patients visiting the ED during weekends had lower enrollment rates than during weekdays (35.4% vs 37.7%). Highest enrollment rates were observed in hospitals with more than 500 beds (43% vs 35.7% with <200 beds), as well as publicly owned hospitals (vs investor vs nonprofit: 42% vs 33.8% vs 36.7%, respectively). Annual enrollment rates notably decreased following the onset of the COVID-19 pandemic (37.8% in January 2020 to 29% in December 2021) (Figure 1). Enrollment was variable across the state, with the highest in Superior California (47.2%) and lowest in Central Coast (27.3%) (Figure 2; eTable 2 in Supplement 1).

In the adjusted model for the ED cohort (Table), risk factors associated with lower odds of 6-month Medicaid enrollment included male sex (compared with female sex: adjusted odds ratio [aOR], 0.74; 95% CI, 0.72-0.76; *P* < .001), Hispanic ethnicity (compared with White: aOR, 0.94; 95% CI, 0.90-0.98; *P* = .007), Spanish speakers (vs English speakers: aOR, 0.72; *P* < .001), no reported race and ethnicity (aOR, 0.04; 95% CI, 0.03-0.04; *P* < .001) or preferred language (aOR, 0.15; 95% CI, 0.14-0.16; *P* < .001), and weekend encounters (vs weekdays: aOR, 0.95; 95% CI, 0.93-0.97; *P* < .001). Factors associated with increased odds of Medicaid enrollment were other race and ethnicity (aOR, 1.12; 95% CI, 1.04-1.22; *P* = .005), age from 27 to 64 years (27-44 years: aOR, 1.04; 95% CI, 1.02-1.06; *P* < .001; 45-64: aOR, 1.32; 95% CI, 1.28-1.37; *P* < .001), and encounters in more than 500 bedsize hospitals (vs < 200 bedsize: aOR, 1.13; 95% CI, 1.00-1.27; *P* = .04) or public hospitals (vs nonprofit: aOR, 1.27; 95% CI, 1.04-1.55; *P* = .02). Compared with patients with a primary diagnosis of infectious diseases, those with dermatologic disorders had lower odds of enrollment, whereas those with conditions including pregnancy, cancer, mental health disorders, cardiovascular or gastrointestinal diseases had higher odds.

**Table.** Logistic Regression of Medicaid Enrollment Rate by 6 Months After Receiving HPE in ED by Patient and Encounter Characteristics

Variable	All patients (N = 585 693)	
	Adjusted OR (95% CI)	*P* value
Encounter year		
2016	1 [Reference]	
2017	1 (0.97-1.04)	.76
2018	1.05 (1.00-1.09)	.04
2019	0.97 (0.92-1.01)	.16
2020	0.81 (0.76-0.86)	<.001
2021	0.88 (0.82-0.95)	.001
Sex		
Female	1 [Reference]	NA
Male	0.74 (0.72-0.76)	<.001
Race and ethnicity		
Alaskan Native or American Indian	0.92 (0.83-1.03)	.13
Asian or Pacific Islander	0.96 (0.89-1.04)	.33
Black	1.07 (1.00-1.14)	.06
Hispanic	0.94 (0.90-0.98)	.007
White	1 [Reference]	NA
Other race and ethnicity^a^	1.12 (1.04-1.22)	.005
No response	0.04 (0.03-0.04)	<.001
Language		
English	1 [Reference]	NA
Asian Language^b^	1.08 (0.96-1.22)	.18
Spanish	0.72 (0.68-0.77)	<.001
Other non-English Language^c^	0.95 (0.83-1.09)	.48
No response	0.15 (0.14-0.16)	<.001
Age, y		
19-26	1 [Reference]	NA
27-44	1.04 (1.02-1.06)	<.001
45-64	1.32 (1.28-1.37)	<.001
Weekend (vs weekday) encounters	0.95 (0.93-0.97)	<.001
Hospital licensed beds		
1-199	1 [Reference]	NA
200-299	1.01 (0.92-1.10)	.89
300-499	1.08 (0.95-1.21)	.27
≥500	1.13 (1.00-1.27)	.04
Hospital ownership		
Nonprofit	1 [Reference]	NA
Investor	1.02 (0.92-1.14)	.68
Public	1.27 (1.04-1.55)	.02
Primary diagnosis^d^		
Chapter 1: Certain Infectious and Parasitic Diseases	1 [Reference]	NA
Chapter 2: Neoplasms	1.37 (1.12-1.68)	.003
Chapter 3: Diseases of the Blood and Blood-Forming Organs and Certain Disorders Involving the Immune System	1.15 (1.04-1.27)	.008
Chapter 4: Endocrine, Nutritional and Metabolic Diseases	1.25 (1.16-1.35)	<.001
Chapter 9: Diseases of the Circulatory System	1.24 (1.16-1.33)	<.001
Chapter 5: Mental and Behavioral Disorders	1.29 (1.21-1.38)	<.001
Chapter 6: Diseases of the Nervous System	1.04 (0.96-1.13)	.30
Chapter 7: Diseases of the Eye and Adnexa	1.05 (0.96-1.14)	.26
Chapter 8: Diseases of the Ear and Mastoid Process	0.97 (0.89-1.06)	.55
Chapter 10: Diseases of the Respiratory System	0.96 (0.90-1.03)	.26
Chapter 11: Diseases of the Digestive System	1.07 (1.01-1.14)	.03
Chapter 12: Diseases of the Skin and Subcutaneous Tissue	0.88 (0.82-0.94)	<.001
Chapter 13: Diseases of the Musculoskeletal System and Connective Tissue	1.04 (0.97-1.11)	.27
Chapter 14: Diseases of the Genitourinary System	1.02 (0.96-1.09)	.55
Chapter 19: Injury, Poisoning and Certain Other Consequences of External Causes	0.95 (0.89-1.01)	.11
Chapter 15: Pregnancy, Childbirth and the Puerperium/Chapter 16: Certain Conditions Originating in the Perinatal Period	2.36 (2.16-2.58)	<.001
Chapter 17: Congenital Malformations, Deformations and Chromosomal Abnormalities	1.57 (0.98-2.51)	.061
Chapter 18: Symptoms, Signs and Abnormal Clinical and Laboratory Findings, Not Elsewhere Specified	1.01 (0.95-1.08)	.69
Chapter 20: External Causes of Morbidity and Mortality	0.9 (0.50-1.62)	.72
Chapter 21: Factors Influencing Health Status and Contact With Health Services	1.08 (1.00-1.18)	.059
Chapter 22: Codes for Special Purposes	0.79 (0.70-0.88)	<.001

Abbreviations: ED, emergency department; HPE, hospital presumptive eligibility; NA, not applicable; OR, odds ratio.

^a^ As defined by the California Department of Health Care Services, including individuals who did not identify with the standard federal race and ethnicity categories.

^b^ As defined by the California Department of Health Care Services, including Cambodian, Cantonese, Hmong, Japanese, Korean, Lao, Mandarin, Mien, other Chinese language, Tagalog, Thai, Vietnamese.

^c^ As defined by the California Department of Health Care Services, including languages not in any other listed categories.

^d^ According to the *International Classification of Diseases, Tenth Revision (ICD-10).*

In comparison, 128 264 of the 206 008 inpatient HPE recipients (62.3%) during the same period enrolled in Medicaid by 6 months (eTable 3 in Supplement 1). Fewer patients in the inpatient cohort did not disclose race and ethnicity or preferred language (31.2% vs 45.7% in the ED cohort). Among those who did disclose, 84.8% enrolled in Medicaid by 6 months (vs 63.6% in the ED cohort).

## Discussion

To our knowledge, this is the first study evaluating 6-month Medicaid enrollment among patients who received HPE in ED. The enrollment rate among the ED cohort was lower than that of the contemporary inpatient cohort. This may be partially explained by different patient characteristics because more patients in the ED did not disclose their race and ethnicity or preferred language. However, even among the patients who did disclose, enrollment rates remained lower. ED patients may have less anticipated postdischarge health care utilization, which can impact perceived needs for ongoing insurance. This represents a missed opportunity, because HPE-approved patients will subsequently lose insurance after 60 days and use ED as their main access point of care again.

In a previous study,^9^ our group demonstrated that Medicaid enrollment was higher among inpatient HPE recipients with longer lengths of stay, likely due to greater touchpoints with staff who could facilitate Medicaid applications. ED HPE enrollees’ lower Medicaid enrollment may therefore be explained by the lack of assistance provided by inpatient hospital personnel. ED patients were likely particularly disadvantaged during periods of sparse staffing on weekends or during hospital surges (the COVID-19 pandemic).^9^ Higher Medicaid enrollment rates in large, publicly-owned hospitals and across certain geographic regions also points to variability in dedicated HPE program resources across the state. This underlines the importance of dedicating additional personnel in the ED and partnering with community stakeholders to continue Medicaid enrollment for patients after ED discharge. The overarching goals of these efforts are to allow a broader patient population to obtain lasting Medicaid coverage through HPE Medicaid and promote appropriate use of ED resources.

### Limitations

This study has several limitations. Medicaid enrollment status does not necessarily indicate lack of health insurance coverage. Patients who switched to alternative types of insurance were not examined in this study, although this was likely the case for a very small group of patients.^10^ In addition, the dataset did not have adequate granularity in demographic information to explain the disproportionately low Medicaid enrollment rate among patients who did not disclose information about their race and ethnicity or preferred language. Finally, because Medicaid is a joint federal-state partnership and its administration can vary considerably across the US, our California-based analyses cannot be generalized to other states.^11^

## Conclusions

This cohort study demonstrated that 37.1% of patients who received HPE in ED enrolled in Medicaid coverage by 6 months. Variability across patient demographics, ED encounter characteristics, and geographic regions in California highlight opportunities to streamline HPE administration and improve staffing assistance to coordinate enrollment in ED. Future directions may also focus on strengthening ED and local partnerships to facilitate Medicaid enrollment and health care utilization after discharge.

## Notes

Supplement 1. eTable 1. Medicaid Enrollment by 6 Months after receiving ED HPE by Patient and Encounter Characteristics eTable 2. Medicaid Enrollment by 6 Months after receiving ED HPE by Geographical Areas in California eTable 3. Comparison between ED versus Inpatient HPE Enrollees in 2016-2021 eTable 4. Multivariable Regression Model Variable List eTable 5. Characteristics of Missing Data in Race/Ethnicity or Language among ED HPE Recipients eFigure: Flowchart of Sample Inclusions and Exclusions

Supplement 2. Data Sharing Statement

## References

[1] Cohen RA. Health Insurance Coverage: Early Release of Quarterly Estimates From the National Health Interview Survey, January 2023–March 2024. Published online 2024. Accessed August 16, 2024. https://www.cdc.gov/nchs/data/nhis/earlyrelease/Quarterly_Estimates_2024_Q11.pdf

[2] Hargraves J, Kennedy K. ER facility prices grew in tandem with faster-growing charges from 2009-2016. Health Care Cost Institute. September 11, 2018. Accessed September 25, 2024. https://healthcostinstitute.org/emergency-room/er-facility-prices-charges-2009-2016

[3] Scott KW, Scott JW, Sabbatini AK, . Assessing catastrophic health expenditures among uninsured people who seek care in US hospital-based emergency departments. JAMA Health Forum. 2021;2(12):e214359. doi:10.1001/jamahealthforum.2021.4359 35977304 PMC8796980

[4] Enrollment Strategies | Medicaid. Accessed September 26, 2024. https://www.medicaid.gov/medicaid/enrollment-strategies/index.html

[5] Winkelman TNA, Segel JE, Davis MM. Medicaid enrollment among previously uninsured Americans and associated outcomes by race/ethnicity-United States, 2008-2014. Health Serv Res. 2019;54(Suppl 1)(suppl 1):297-306. doi:10.1111/1475-6773.13085 30394525 PMC6341200

[6] Knowlton LM, Tran LD, Arnow K, . Emergency Medicaid programs may be an effective means of providing sustained insurance among trauma patients: A statewide longitudinal analysis. J Trauma Acute Care Surg. 2023;94(1):53-60. doi:10.1097/TA.0000000000003796 36138539 PMC9805493

[7] Knowlton LM, Logan DS, Arnow K, . Do hospital-based emergency Medicaid programs benefit trauma centers? a mixed-methods analysis. J Trauma Acute Care Surg. 2024;96(1):44-53. doi:10.1097/TA.0000000000004162 37828656 PMC10841404

[8] Handley TJ, Boncompagni AC, Arnow K, . Evaluating emergency Medicaid program policy changes during the COVID-19 pandemic. J Surg Res. 2023;289:97-105. doi:10.1016/j.jss.2023.03.030 37086602 PMC10043965

[9] Knowlton LM, Arnow K, Trickey AW, . Hospital presumptive eligibility emergency Medicaid programs: an opportunity for continuous insurance coverage? Med Care. 2024;62(9):567-574. doi:10.1097/MLR.0000000000002026 38986116 PMC11315624

[10] Glied SA, Jackson A. Who entered and exited the individual health insurance market before and after the Affordable Care Act? evidence from the Medical Expenditure Panel Survey. Issue Brief (Commonw Fund). 2018;2018:1-11. 30497127

[11] Donohue JM, Cole ES, James CV, Jarlenski M, Michener JD, Roberts ET. The US Medicaid program: coverage, financing, reforms, and implications for health equity. JAMA. 2022;328(11):1085-1099. doi:10.1001/jama.2022.14791 36125468

