# Supplementary material for: Medicaid Enrollment After Hospital Presumptive Eligibility in the Emergency Department
Source: JAMA Health Forum. 2025 Apr 25;6(4):e250768. doi: 10.1001/jamahealthforum.2025.0768 (PMC12032561; doi:10.1001/jamahealthforum.2025.0768)
Supplement: Supplement 1. — eTable 1. Medicaid Enrollment by 6 Months after receiving ED HPE by Patient and Encounter Characteristics eTable 2. Medicaid Enrollment by 6 Months after receiving ED HPE by Geographical Areas in California eTable 3. Comparison between ED versus Inpatient HPE Enrollees in 2016-2021 eTable 4. Multivariable Regression Model Variable List eTable 5. Characteristics of Missing Data in Race/Ethnicity or Language among ED HPE Recipients eFigure: Flowchart of Sample Inclusions and Exclusions [file jamahealthforum-e250768-s001.pdf]

Supplemental Online Content

Wang S, Arnow K, Sakamoto MM, Knowlton LM. Medicaid enrollment after hospital presumptive eligibility in the emergency department. *JAMA Health Forum*. 2025;6(4):e250768. doi:10.1001/jamahealthforum.2025.0768

eTable 1. Medicaid Enrollment by 6 Months after receiving ED HPE by Patient and Encounter Characteristics

eTable 2. Medicaid Enrollment by 6 Months after receiving ED HPE by Geographical Areas in California

eTable 3. Comparison between ED versus Inpatient HPE Enrollees in 2016-2021 **e**

eTable 4. Multivariable Regression Model Variable List

eTable 5. Characteristics of Missing Data in Race/Ethnicity or Language among ED HPE Recipients

eFigure: Flowchart of Sample Inclusions and Exclusions

This supplemental material has been provided by the authors to give readers additional information about their work.

© 2025 Wang S et al. *JAMA Health Forum*.

eTable 1: Medicaid Enrollment by 6 Months after Receiving ED HPE by Patient and Encounter Characteristics

	N (Row %)			*P*
	**Did Not Enroll in Medicaid (n = 368,263)**	**Enrolled in Medicaid (n = 217,430)**	**Total (n = 585,693)**	
**Year**				<.001
2016	54,231 (59.4)	36,996 (40.6)	91,227	
2017	61,015 (59.9)	40,924 (40.1)	101,939	
2018	64,522 (60.6)	41,876 (39.4)	106,398	
2019	64,387 (61.5)	40,363 (38.5)	104,750	
2020	64,659 (66.9)	32,050 (33.1)	96,709	
2021	59,449 (70.2)	25,221 (29.8)	84,670	
**Sex**				<.001
Female	141,921 (57)	107,013 (43)	248,934	
Male	226,342 (67.2)	110,417 (32.8)	336,759	
**Ethnicity**				<.001
White	26,143 (35.6)	47,375 (64.4)	73,518	
Hispanic	69,205 (39.4)	106,290 (60.6)	175,495	
Black	11,479 (33.9)	22,350 (66.1)	33,829	
Asian or Pacific Islander	4,546 (35.4)	8,278 (64.6)	12,824	
Alaskan Native or American Indian	623 (37)	1,062 (63)	1,685	
Other Race/Ethnicity	9,095 (32.9)	18,515 (67.1)	27,610	
No Response	247,172 (94.8)	13,560 (5.2)	260,732	
**Language**				<.001
English	201,392 (54.4)	168,751 (45.6)	370,143	
Spanish	116,473 (72.4)	44,337 (27.6)	160,810	
Asian Language	1,497 (58.1)	1,078 (41.9)	2,575	
Other Non-English Language	2,406 (63.8)	1,368 (36.2)	3,774	
No Response	46,495 (96.1)	1,896 (3.9)	48,391	
**Age**				<.001
19-26	94,545 (60.2)	62,408 (39.8)	156,953	
27-44	176,002 (64.7)	96,156 (35.3)	272,158	
45-64	97,716 (62.4)	58,866 (37.6)	156,582	
**Hospital ownership**				<.001
Public	94,545 (58)	43,155 (42.0)	102,731	
Investor	176,002 (66.2)	36,881 (33.8)	109,012	
Non-Profit	97,716 (63.3)	137,394 (36.7)	373,950	
**Hospital bed size**				<.001
1-199	102,562 (64.3)	56,933 (35.7%)	159,495	
200-299	61,977 (64.5)	34,099 (35.5%)	96,076	
300-499	157,045 (63.3)	91,221 (36.7%)	248,266	
500+	46,679 (57)	35,177 (43.0%)	81,856	
**Discharge day**				<.001
Weekday	273,033 (62.3)	165,302 (37.7%)	438,335	
Weekend	95,230 (64.6)	52,128 (35.4%)	147,358	

ED: emergency department; **HPE**: Hospital Presumptive Eligibility

© 2025 Wang S et al. *JAMA Health Forum*.

eTable 2: Medicaid Enrollment by 6 Months after receiving ED HPE by Geographical Areas in California

	Did Not Enroll in Medicaid (n = 368,241)		Enroll in Medicaid (n = 217,370)		Total (n = 585,611)
	N	%	N	%	N
**Region**					
Superior California	20,210	52.8	18,068	47.2	38,278
North Coast	3,248	62.5	1,948	37.5	5,196
San Francisco Bay Area	30,422	56.3	23,574	43.7	53,996
North San Joaquin Valley	14,101	58.9	9,857	41.1	23,958
Central Coast	27,609	72.7	10,370	27.3	37,979
South San Joaquin Valley	27,201	60.7	17,611	39.3	44,812
Inland Empire	60,858	62.2	37,025	37.8	97,883
Los Angeles County	121,277	67.3	58,865	32.7	180,142
Orange County	31,153	68.3	14,476	31.7	45,629
San Diego-Imperial	32,162	55.7	25,576	44.3	57,738

ED: emergency department; **HPE**: Hospital Presumptive Eligibility

eTable 3: Comparison between ED versus Inpatient HPE Enrollees in 2016-2021

	ED (N= 585,693)		Inpatient (N= 206,008)		
	n	%	*n*	%	***P*-value**
Overall 6-Month Medicaid Enrollment	217,430	37.1	128,246	62.3	<.001
No Response to the Question of Race/Ethnicity or Preferred Language	267,721	45.7	64,435	31.2	<.001
6-Month Medicaid Enrollment among Those with Race/Ethnicity and Preferred Language Information	202,333 out of 317,972 (63.6%)		120,082 out of 141573 (84.8%)		<.001

ED: emergency department; **HPE**: Hospital Presumptive Eligibility

© 2025 Wang S et al. *JAMA Health Forum*.

eTable 4: Multivariable Regression Model Variable List

Variable Name	*Type*	*Values*
Encounter Year	Categorical	2016, 2017, 2018, 2019, 2020, 2021
Male sex	Binary	0, 1
Race/ethnicity	Categorical	White, Hispanic, Black, Asian or Pacific Islander, Alaskan Native or American Indian, other race/ethnicity, non response
Language	Categorical	English, Spanish, Asian language, other non-English language, no response
Age	Categorical	19-26, 27-44, 45-64
Weekend Encounters	Binary	0, 1
Hospital licensed beds	Categorical	1-199, 200-299, 300-499, 500+
Hospital ownership	Categorical	non-profit, investor, public
Primary Diagnosis (ICD Chapter)	Categorical	Chapter I - Certain infectious and parasitic diseases, Chapter II – Neoplasms, Chapter III - Diseases of the blood and blood-forming organs and certain disorders involving the immune system, Chapter IV - Endocrine, nutritional and metabolic diseases, Chapter IX - Diseases of the circulatory system, Chapter V - Mental and behavioural disorders, Chapter VI - Diseases of the nervous system, Chapter VII - Diseases of the eye and adnexa, Chapter VIII - Diseases of the ear and mastoid process, Chapter X - Diseases of the respiratory system, Chapter XI - Diseases of the digestive system, Chapter XII - Diseases of the skin and subcutaneous tissue, Chapter XIII - Diseases of the musculoskeletal system and connective tissue, Chapter XIV - Diseases of the genitourinary system, Chapter XIX - Injury, poisoning and certain other consequences of external causes, Chapter XV - Pregnancy, childbirth and the puerperium/Chapter XVI - Certain conditions originating in the perinatal period, Chapter XVII - Congenital malformations, deformations and chromosomal abnormalities, Chapter XVIII - Symptoms, signs and abnormal clinical and laboratory findings, not elsewhere specified, Chapter XX - External causes of morbidity and mortality, Chapter XXI - Factors influencing health status and contact with health services, Chapter XXII - Codes for special purposes

ICD: International Classification of Diseases

© 2025 Wang S et al. *JAMA Health Forum*.

eTable 5: Characteristics of Missing Data in Race/Ethnicity or Language among ED HPE Recipients

	Total	Missing Data in Race/Ethnicity or Language	
	N	***n***	***%***
**2016**	91,227	37,639	41.3
**2017**	101,939	42,750	41.9
**2018**	106,398	46,903	44.8
**2019**	104,750	45,737	43.7
**2020**	96,709	46,829	48.4
**2021**	84,670	47,863	56.5

**All ED HPE Recipients (2016-2021)**		**Missing Data in Language**	
		No	Yes
**Missing Data in Race/Ethnicity**	No	317,972	6,989
	Yes	219,330	41,402

ED: emergency department; **HPE**: Hospital Presumptive Eligibility

© 2025 Wang S et al. *JAMA Health Forum*.

eFigure: Flowchart of Sample Inclusions and Exclusions

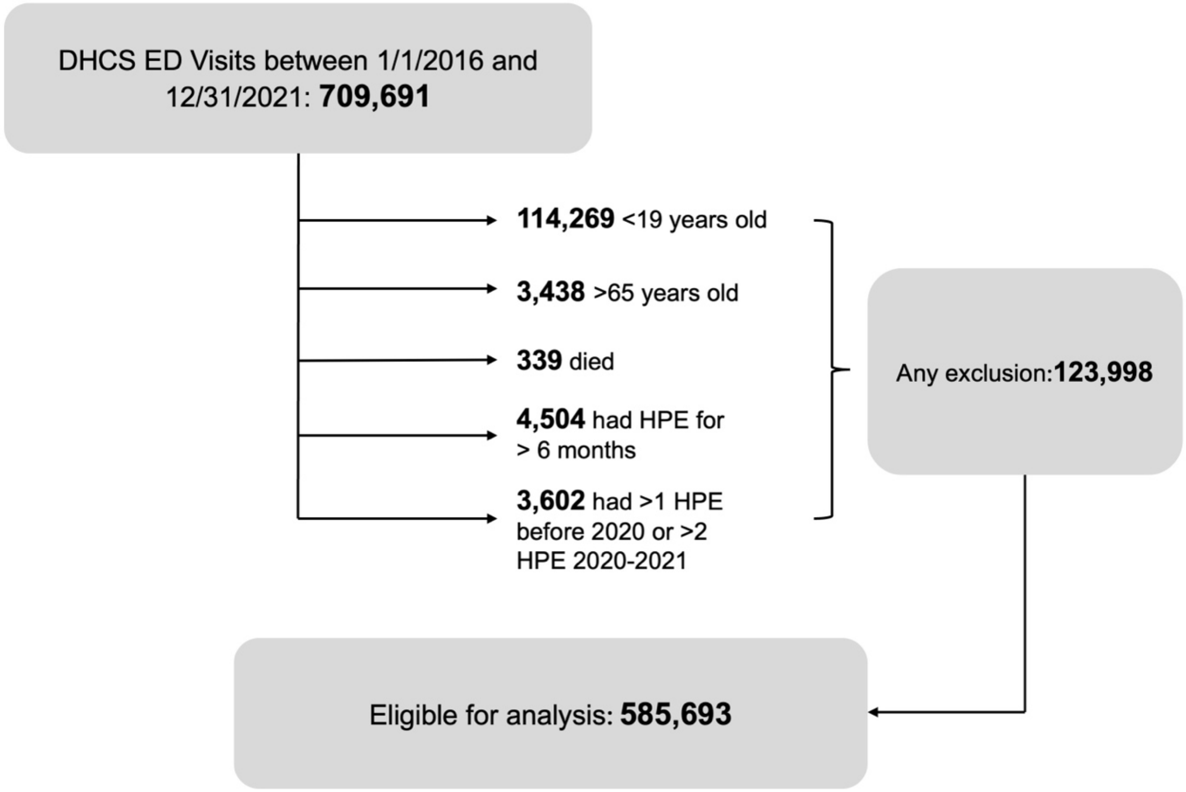

DHCS: Department of Health Care Services; **ED**: emergency department; **HPE**: Hospital Presumptive Eligibility

© 2025 Wang S et al. *JAMA Health Forum*.
