# Supplementary material for: Medicaid Enrollment After Hospital Presumptive Eligibility in the Emergency Department
Source: JAMA Health Forum. 2025 Apr 25;6(4):e250768. doi: 10.1001/jamahealthforum.2025.0768 (PMC12032561; doi:10.1001/jamahealthforum.2025.0768)
Supplement: Supplement 2. — Data Sharing Statement [file jamahealthforum-e250768-s002.pdf]

Data Sharing Statement

Wang. Medicaid Enrollment After Hospital Presumptive Eligibility in the Emergency Department. *JAMA Health Forum*. Published April 25, 2025.
doi:10.1001/jamahealthforum.2025.0768

Data
Data available: Yes
Data types: Data (not involving human participants), Data dictionary
How to access data: drlmk@stanford.edu
When available: With publication

Supporting Documents
Document types: None

Additional Information
Who can access the data: Researchers whose proposed use of the data has been approved
Types of analyses: Specified purpose per discussion with authors
Mechanisms of data availability: After approval of a proposal
